# Appendix A Overview of National Population Health and Canadian Community Health Surveys*

**DOI:** 10.1186/1472-6874-4-S1-S35

**Published:** 2004-08-25

**Authors:** Marie Desmeules

**Affiliations:** 1 Centre for Chronic Disease Prevention and Control, Health Canada, 120 Colonnade Rd, Ottawa, Canada

## National Population Health Survey

### General

The National Population Health Survey (NPHS) was designed to provide information related to the health of Canadians. Statistics Canada first conducted the NPHS in 1994 and continued every second year thereafter. The goal was to improve the information available to support the development and evaluation of health policies in Canada. The survey produces both cross-sectional information and longitudinal data in two-year intervals.

### Methodology

The target population of the NPHS includes household residents from the 10 provinces, with the principal exclusion of populations on Indian reserves, Canadian Forces Bases and some remote areas in Quebec and Ontario. Separate surveys were conducted to cover the Yukon and Northwest Territories and institutions (long-term residents expected to stay longer than six months in health care institutions with four beds or more in all provinces). A representative sample of the Canadian population was obtained with household proportions based on 1991 Census data. The samples from each province were proportional to the province's 1991 Census population size.

**Figure 1:**
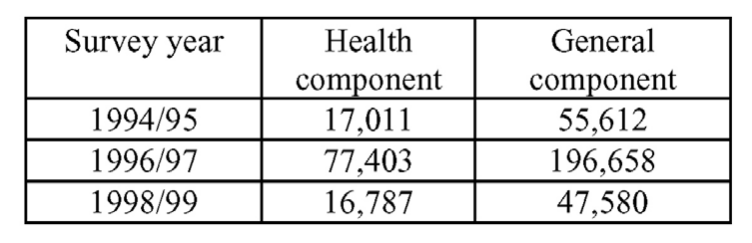
Sample sizes

Within each household, limited information on all members in the household was collected by proxy and this information made up the data found within the *general component *for all cycles. The *health component *consists of data gathered from an in-depth interview with a randomly selected member of the household (12 years and over for the 1994–1995 cycle and all ages for the subsequent cycles). The sample sizes from each cycle and each component are in Figure 1.

The large difference in the sample size for cycle 2 compared to the other two cycles is a result of large provincial buy-ins from Ontario, Alberta and Manitoba for 1996–1997.

The core content of the questionnaire includes information on health status (self-perception of health, functional ability, chronic conditions, activity restrictions); use of health services (visits to health care providers, hospital care and drug use); risk factors (smoking, alcohol use, physical activity); and demographic and socio-economic status (age, sex, education, ethnicity, household income, labour force status).

All cycles contain the same core content; however, the focus content differs from cycle to cycle, with 1994–1995 focus content investigating psycho-social health, and 1996–1997 focusing on access to health services.

### Data Collection

Data from all cycles of the NPHS were collected through a combination of in-person interviews as well as telephone interviews (the percentage of each method varied for each cycle). The cross-sectional component of the survey, 1994–1995 and 1998–1999 cycles, selected a total of 27,263 and 19,973 households respectively, with corresponding overall household response rates of 89% and 88%. The 1996–1997 cycle differed from the other two cycles in that it allowed buy-in samples in Alberta, Manitoba and Ontario. For the 1996–1997 cycle, there was a total household sample of 95,370, with a household response rate of 83%.

## Canadian Community Health Survey

### General

The Canadian Community Health Survey (CCHS) is part of an initiative to provide health information at the regional and provincial levels. In 2000, Statistics Canada began collecting data for the CCHS. The CCHS consists of two cross-sectional surveys conducted over two years, on a repeating cycle. The first survey (cycle 1.1) was designed to provide data at the health region level while the second survey (cycle 1.2) will focus on a specific health topic, providing provincial-level data.

### Methodology

The CCHS targets individuals 12 years of age and older who live in private dwellings. People living on Indian reserves or Crown lands, residents of institutions, full-time members of the Canadian Armed Forces and residents of certain remote regions were excluded. The data from the three Territories were not included in the first half of cycle 1.1 because data collection for these areas began later than the rest of the country.

For the collection of regional-level data, each province was divided into health regions and each Territory was designated as a single health region. With some minor changes, the boundaries used for the health regions correspond to the geography of the 1996 Census. There were a total of 136 health regions in Canada.

Allocation of sample size to the provinces and territories was distributed according to their respective populations and the number of health regions they contained. Also, each province's sample was allocated among its health regions proportionally to the square root of the estimated population in each health region.

### Data Collection

An important goal of the CCHS was to collect health region-relevant data. To accomplish this, the questionnaire was divided into two parts: a common content section and an optional content section containing questions selected to meet the needs of each health region.

The common content portion of the survey provided data on the general health of the respondent, health services utilization, alcohol and tobacco use, and screening tests. Optional content data mainly examined psychological issues and secondary prevention.

Respondents were chosen by either a multi-stage stratified cluster design or by random digit dialing. Selection of individual respondents was designed to ensure overrepresentation of youth and seniors. To do this, two people were randomly chosen and interviewed in a small percentage of households selected while one was chosen from the remainder. Each respondent was assigned a weight to represent their contribution to the total population.

The questionnaire was administered via computer-assisted personal interviews and telephone interviews. In cases where the intended respondent was not available, a proxy interview was administered. In total, 130,000 individuals were sampled for the health region-level survey, while 30,000 were sampled for the provincial-level survey.

## Health Services Access Survey

### General

The Health Services Access Survey (HSAS) was conducted in 2001 by Statistics Canada and is a supplement to the 2000 CCHS. The goal of this survey was to provide information on the health care service experience of respondents using selected health care services. The two main topic areas were access to basic health care and waiting times for specialized services of incident conditions.

### Methodology

The data obtained from this survey can be linked to the CCHS through two main administrative variables. The sample used for the HSAS was a sub-sample of the CCHS sample. Only one person per household could be selected and the inclusion criteria included being at least 15 years of age by the specified date, agreeing to allow sharing of their CCHS data with the provincial partners, and providing a telephone number where they could be reached. No respondents were selected from the Territories. There was an overall sample size of 14,210 for this survey.

### Analysis

Data presented from these various sources have been calculated using a weighted approach so that estimates reflect the national population. Confidence intervals have been calculated using the Bootstrap method.

## Note

*The views expressed in this report do not necessarily represent the views of the Canadian Population Health Initiative, the Canadian Institute for Health Information or Health Canada.

